# Medical students as adverse drug event managers, learning about side effects while improving their reporting in clinical practice

**DOI:** 10.1007/s00210-021-02060-y

**Published:** 2021-03-05

**Authors:** M. Reumerman, J. Tichelaar, M.C. Richir, M.A. van Agtmael

**Affiliations:** 1 Pharmacotherapy Section, Department of Internal Medicine, Amsterdam UMC, location VU University Medical Center, De Boelelaan 1117, 1081 HV Amsterdam, The Netherlands; 2 Research and Expertise Center in Pharmacotherapy Education (RECIPE), De Boelelaan 1117, 1081 HV Amsterdam, The Netherlands

**Keywords:** Medical education, Clinical pharmacology, Pharmacotherapy, Pharmacovigilance, Reporting ADRs

## Abstract

Managing adverse drug reactions (ADRs) is a challenge, especially because most healthcare professionals are insufficiently trained for this task. Since context-based clinical pharmacovigilance training has proven effective, we assessed the feasibility and effect of a creating a team of Junior-Adverse Drug Event Managers (J-ADEMs). The J-ADEM team consisted of medical students (1st–6th year) tasked with managing and reporting ADRs in hospitalized patients. Feasibility was evaluated using questionnaires. Student competence in reporting ADRs was evaluated using a case-control design and questionnaires before and after J-ADEM program participation. From Augustus 2018 to Augustus 2019, 41 students participated in a J-ADEM team and screened 136 patients and submitted 65 ADRs reports to the Netherlands Pharmacovigilance Center Lareb. Almost all patients (*n* = 61) found it important that “their” ADR was reported, and all (*n* = 62) patients felt they were taken seriously by the J-ADEM team. Although attending physicians agreed that the ADRs should have been reported, they did not do so themselves mainly because of a “lack of knowledge and attitudes” (50%) and “excuses made by healthcare professionals” (49%). J-ADEM team students were significantly more competent than control students in managing ADRs and correctly applying all steps for diagnosing ADRs (control group 38.5% vs. intervention group 83.3%, *p* < 0.001). The J-ADEM team is a feasible approach for detecting and managing ADRs in hospital. Patients were satisfied with the care provided, physicians were supported in their ADR reporting obligations, and students acquired relevant basic and clinical pharmacovigilance skills and knowledge, making it a win-win-win intervention.

**Supplementary Information:**

The online version contains supplementary material available at 10.1007/s00210-021-02060-y.

## Introduction


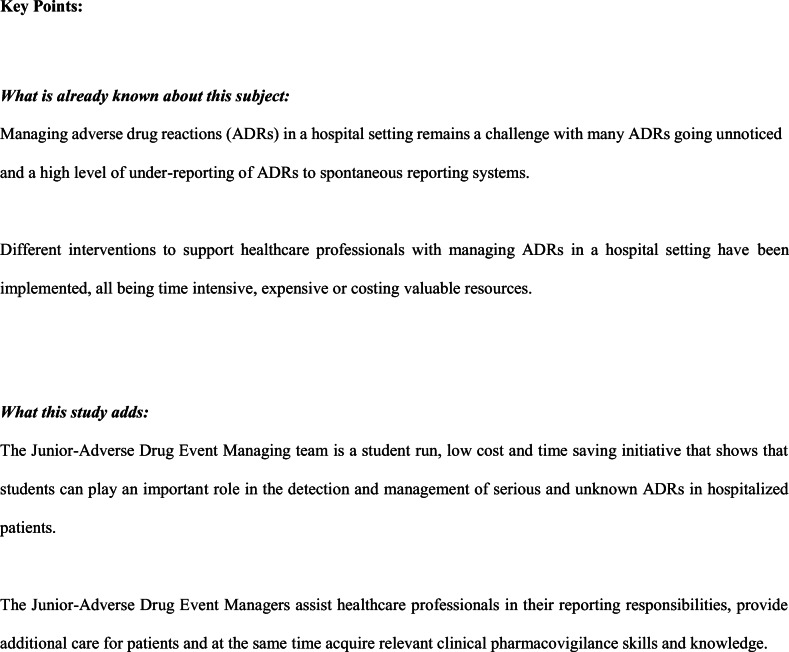
Managing adverse drug reactions (ADRs) remains a challenge given the increasing complexity of therapeutics, the aging population, and the growing number of patients with multimorbidity and polypharmacy (Coleman and Pontefract 2016). Previous studies have shown that 3.5–10% of patients visiting the emergency department experience an ADR (Bouvy et al. 2015; Roulet et al. 2012; Budnitz et al. 2006), that more than half of all ADRs go unnoticed upon hospital admission (Dormann et al. 2003; Roulet et al. 2013), and that the estimated median underreporting rate is 94% (Hazell and Shakir 2006). While detecting and reporting ADRs is important for patient safety at an individual and population level, many ADR reports are of poor quality (Hazell and Shakir 2006; Miguel et al. 2013; Lopez-Gonzalez et al. 2014). Multiple determinants for low ADR detection and reporting rates have been found, most of which are related to the awareness and pharmacological knowledge of attending healthcare professionals (Dormann et al. 2003; Hazell and Shakir 2006; Lopez-Gonzalez and Figueiras 2009).

While most healthcare professionals recognize the importance of ADR management, they lack the skills and knowledge to do so (Pagotto et al. 2013). This is not surprising since healthcare students receive almost no education on this topic, and the educational activities that do exist are mainly outdated and lecture based (Reumerman et al. 2018; Jenny Hartman 2017; Schutte et al. 2017a) and few have clinical and or long-term effects (Reumerman et al. 2020a; Arici et al. 2015). Context-based clinical pharmacovigilance training, such as reporting ADRs in clinical practice (Reumerman et al. 2018; Reumerman et al. 2020a; Schutte et al. 2018a; Sullivan and Spooner 2008) or assessing real ADR reports (Schutte et al. 2017b), has proven effective in increasing students’ pharmacovigilance skills and knowledge (Reumerman et al. 2018).

While context-based clinical pharmacovigilance educational interventions are effective, they often do not accurately reflect the future work experience of medical students, which limits their educational potential (Schutte et al. 2017b; Schutte et al. 2017c). To maximize the educational value of these interventions, we set up a team of Junior-Adverse Drug Event Managers (J-ADEM), based on a successful intervention from Denmark called the Adverse Drug Event Manager (ADEM) (Sørup et al. 2015; Vinther et al. 2017), whereby a first year resident in clinical pharmacology assist physicians in reporting already detected ADRs. The J-ADEM team not only had similar responsibilities but also actively screened for previously unrecognized ADRs. Since the J-ADEM team was incorporated in the Learner-Centered Student-Run Clinic (LC-SRC) of the VUmc, it is also completely run by medical students (Schutte et al. 2018b; Schutte et al. 2018c).

The aim of this study was to analyze the feasibility and educational value of the J-ADEM program in a tertiary academic hospital and analyze the educational value of such a program in terms of students’ attitudes, skills, and knowledge in pharmacovigilance.

## Methods

This prospective observational study was set up to evaluate the feasibility of a J-ADEM team in a tertiary academic hospital. The J-ADEM team was developed to systematically screen patients hospitalized for a suspected serious or unknown ADR or who developed a serious or unknown ADR while in hospital.

### Setting and population

The Amsterdam UMC, location VUmc, is a tertiary academic hospital that provides specialty care and has about 700 beds on 23 different wards. In August 2018, the J-ADEM team started on two wards (internal medicine and ear, nose, and throat) and also worked in the outpatient clinics of these specialties. All staff (physicians, pharmacist, nurses, and paramedics) were able to contact the J-ADEM team (by phone or email) if they suspected an ADR in a patient. The J-ADEMs assessed all such patients aged 18 years or older; patients with either deliberate or unintentional overdose were excluded from the study.

### The J-ADEM team and procedure

Each week, the J-ADEM team is managed by a variable team of two medical students (1st–6th year) who participate voluntarily as part of the LC-SRC. The LC-SRC is an extracurricular program (Schutte et al. 2017c; Schutte et al. 2018b) dedicated to pharmacotherapy and medication safety initiatives with multiple projects focusing on cardiovascular risk management (Schutte et al. 2018c), medication review (Reumerman et al. 2020b), and ADR report assessment (Schutte et al. 2017b). Students who participate in the LC-SRC program are assigned to one or more projects on a weekly basis based on their availability.

**Fig. 1:**
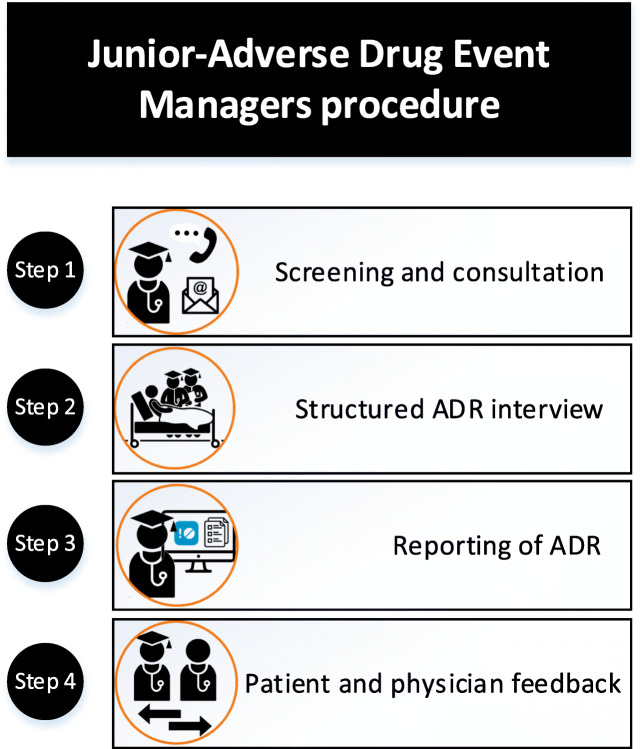
Junior-Adverse Drug Event Managers procedure. The first step consisted of identifying all patients with potential ADRs by screening or being consulted by a healthcare professional. The second step consisted of reviewing the patient’s electronic patient record (EPR) and performing a thorough medication and side effect interview with the patient. The third step consisted of reporting the ADR to the Netherlands Pharmacovigilance Center Lareb and handling all follow-up questions. The final step consisted of providing the attending physician with feedback received from Lareb and uploading this information into the patient’s EPR

The J-ADEM team procedure consisted of four consecutive steps (Fig. 1), which were all performed by all of the students. The first step was identifying all patients with potential ADRs on the two wards. This was done in two ways: either healthcare professionals could report suspected ADRs to the team by providing the patient’s initials, personal identification number, and short description of the suspected ADR or J-ADEM team screened the records of patients admitted to the two wards for suspected ADRs. The second step consisted of reviewing the patient’s electronic patient record (EPR) and performing a thorough medication and side effect interview with the patient. With this information, the J-ADEM team completed four essential steps for diagnosing a possible ADR, using the BAT-M method (Pirmohamed et al. 2004). (Coleman and Pontefract 2016) The symptoms were consistent with the known adverse effect profile of the drug (according to the Dutch National Formulary). (Bouvy et al. 2015) There was a temporal relationship between the start of drug therapy and ADR onset. (Roulet et al. 2012) Appropriate investigations excluded other causes. (Budnitz et al. 2006) The pharmacological mechanism of action underlying the ADR could be explained. All patients considered to have an ADR by the students were discussed with a clinical pharmacologist (MR) and the attending physician (if present), to establish whether the patient was experiencing an ADR. Thereafter, the students reported the ADR to the Netherlands Pharmacovigilance Center Lareb and handled all follow-up questions. Students also wrote a note in the EPR, updated any allergy information (if relevant), and uploaded the ADR report form into the EPR. The final step consisted of providing the attending physician with feedback received from Lareb and uploading this information into the patient’s EPR.

### Evaluation instruments

All three parties (students, attending physician, and patients hospitalized with an ADR) involved in this study completed relevant questionnaires.

All students participating in the LC-SRC were asked to fill in a preintervention and postintervention e-questionnaire, using Castor EDC. Medical students who participated at least once in the J-ADEMs team were included in the intervention group, and medical students who participated in other LC-SRC activities but not in the J-ADEMs team were included as a control group. This e-questionnaire consisted of three parts: baseline characteristics, intention/attitudes, and knowledge/skills (16 questions). Answers were given on a 5-point Likert scale. The preintervention e-questionnaire was sent to both the control and intervention group 4 weeks before the first J-ADEM started (July 2018). The postintervention e-questionnaire was sent to both groups 1 week after the inclusion period ended (August 2019). If students did not respond, two reminders were sent at two-weekly intervals.

All physicians who attended a patient with a medication-related problem and who did not report the suspected ADR were asked two questions: (Coleman and Pontefract 2016) “Do you think it is relevant to report this suspected ADR to Lareb?” and if they agreed that it was relevant: (Bouvy et al. 2015) “Why did you not report the suspected ADR or notify it to the J-ADEM?”.

All patients who had been interviewed about medications and side effects by the J-ADEM were asked to fill in a printed questionnaire, consisting of two parts: (Coleman and Pontefract 2016) relevance and motivation for reporting serious ADRs and (Bouvy et al. 2015) evaluation of the J-ADEM team (11 questions). Answers were given on a 5-point Likert scale.

#### Ethical aspects

This study did not fall under the scope of the Dutch Medical Research Involving Human Subjects Act (reference number 2018.097). Physician and patient participation was voluntary and based on informed consent. The ethics review board of the Netherlands Association for Medical Education (NVMO) reviewed the protocol regarding the students’ participation and approved this study (ID: 826).

#### Data analysis

All data were imported in SPSS Statistics 22 (IBM Corp.; Armonk, New York). Descriptive statistics were used to report frequencies and medians/interquartile range (IQR) of survey results. Differences in competence scores between intervention and control groups were compared using Mann-Whitney *U* test (alpha 0.5 and *p* < 0.05). Categorical variables were calculated using chi-square test (alpha 0.5 and *p* < 0.05).

## Results

From Augustus 2018 through Augustus 2019, 136 patients were screened for medication-related problems, and 65 high-quality ADRs reports, of which 50 ADRs (77%) were classified as severe reactions, were submitted to the Netherlands Pharmacovigilance Center Lareb by the J-ADEM team. These ADR reports included a serious gastro-intestinal bleed because of acenocoumarol, hypokalemia because of anidulafungin, and even a Stevens-Johnson syndrome because of trimethoprim-sulfamethoxazole.

### Patient attitudes toward ADR reporting

Sixty-two (86%) patients returned the questionnaire, 61 of whom (98%) found it (extremely) relevant to report an ADR to Lareb if the suspected ADR led to hospital admission. Their main motive was that it would prevent other patients from having a similar reaction (median 5, IQR 5–5) and increase medication safety (median 5, IQR 5–5). Receiving a personal feedback letter (median 3, IQR 2–4) and raising general awareness (median 4, IQR 3–4) about that ADR were considered less important reasons (Table 1).

**Table 1:** Patient attitudes and Junior-Adverse Drug Event Manager (J-ADEM) team evaluations

	*N*	Median (IQR)	Extremely irrelevant	Irrelevant	Neither relevant nor irrelevant	Relevant	Extremely relevant
Patient attitudes to ADR reporting							
How relevant is an ADR report, when the suspected ADR leads to a hospital admission.	62	5 (5–5)	-	-	1	14	47
How important are underlying motives for you to report an ADR …							
Prevent others from a similar reaction	62	5 (5–5)	-	-	-	12	50
To increase medication safety	62	5 (5–5)	-	-	-	15	47
Receive a personal feedback letter	62	3 (2–4)	12	14	12	11	13
Increase general awareness regarding this ADR	62	4 (3–4)	-	6	12	29	15
That healthcare professionals can learn from this ADR	62	5 (4–5)	-	5	7	18	32
	*N*	Median (IQR)	Insufficient	Dubious	Sufficient	Good	Excellent
Patient evaluations of the J-ADEM team							
What is your opinion regarding the …							
Professional behavior of the students (in comparison with a medical doctor) ?	62	5 (4–5)	-	-	5	15	42
Information received form the students (possibility to ask questions, answers given to you) ?	62	4.5 (4–5)	-	2	8	21	31
Feeling comfortable with the students during this consultation?	62	5 (4–5)	-	-	4	16	42
Feeling of being taken seriously by the students?	62	5 (4–5)	-	-	-	16	46
	*N*	Median (IQR)	Definitely not	Probably not	Unsure	Probably	Definitely
Would you again agree to a student ADR interview?	62	5 (4–5)	-	-	2	23	37

Upper part: patients (hospitalized with an ADR) attitudes to report ADRs. Lower part: patients (hospitalized with an ADR) evaluations of the J-ADEM team.

### J-ADEM patient evaluations

Overall, patient satisfaction was high, with all patients feeling that they had been taken seriously; 60 patients (97%) would agree to another J-ADEM consultation, and 52 patients (84%) said that they had received good or excellent information (Table 1).

### Physician outcomes

All ADRs detected by the J-ADEM teams were reported to Lareb and were considered by the attending physicians to be relevant to report. In 56 (out of 65) cases (86%), the attending physicians answered the question why they had not reported the ADR themselves, providing in total 146 reasons (average 2.6 reasons per physician). “Factors associated with ADR-related knowledge and attitudes” (*n* = 73; 50.0%) and “Excuses made by professionals” (*n* = 72; 49.3%) were the reasons mentioned the most often (Table 2).

**Table 2:** Physician reasons not to report adverse drug reactions (ADRs)

Determinants	Frequency	Percentage
Attitudes relating to professional activity	1	0.7%
Financial incentives	0	-
Litigation concerns	0	-
Ambition to publish	1	0.7%
Factors associated with ADR-related knowledge and attitudes	73	50%
Complacency *(only safe medications are marketed)*	5	3.4%
Insecurity *(determining whether or not a drug is responsible for a particular ADR)*	6	4.1%
Diffidence *(fear of appearing ridiculous)*	5	3.4%
Indifference *(contributing to the general advancement of medical knowledge/lack of understanding of the purpose of reporting)*	33	22.6%
Ignorance *(only severe ADRs need to be reported)*	24	16.4%
Excuses made by professionals	72	49.3%
Lack of time	52	35.6%
Different care priorities	5	3.4%
Difficulty in accessing report form	3	2.1%
Reporting process as extremely bureaucratic and complex	12	8.2%
Aversion to disclosing confidential information	0	-

Physician reasons not to report adverse drug reactions to the Netherlands Pharmacovigilance left Lareb subdivided into known determinants influencing the adverse drug reaction (ADR) reporting rates in healthcare professionals.

### Student outcomes

Forty-one students participated, performing on average 5.2 consultations per student (range 1–9 times). Medical students who participated in other LC-SRC activities but not in the J-ADEMs team were included as a control group. In total, 32 students (65%) in the control group and 36 students (88%) in the intervention group filled in the e-questionnaire (Table 3 and Supplementary Table S1).

**Table 3:** Student intentions, attitudes, and knowledge regarding adverse drug reaction (ADR) reporting

	Preintervention test			Postintervention test			Preintervention vs. postintervention test	
	Control group (32)	Intervention group (36)	*p* value	Control group (31)	Intervention group (32)	*p* value	Control group	Intervention group
I intend to report serious ADRs that I encounter to the competent authority.^a^	4 (3–5)	4 (3–5)	0.331	4 (3–5)	5 (4–5)	**0.024**	0.427	**0.009**
I intend to report new ADRs that I encounter to the competent authority.^a^	4 (3–5)	4 (3–5)	0.541	4 (3–5)	5 (4–5)	**0.003**	0.354	**0.006**
I intend to report all ADRs that I encounter to the competent authority.^a^	4 (2–5)	4 (2–5)	0.602	4 (3–5)	3 (2–4)	**0.044**	0.460	0.102
How likely do you think the following outcomes will be if you report an ADR?								
Contributes to the safe use of medicines^a^	4 (3–5)	4 (3–5)	0.389	4 (3–5)	4 (4–5)	**0.031**	0.652	**<0.001**
Improves patient safety^a^	4 (3–5)	4 (3–5)	0.964	4 (3–5)	5 (4–5)	**<0.001**	0.225	**<0.001**
Educates others about drug risks^a^	4 (3–5)	3 (3–4)	0.209	4 (3–5)	5 (3–5)	**0.032**	**0.035**	**<0.001**
Personally beneficial^a^	3 (2–4)	3 (2–4)	0.463	4 (2–4)	4 (3–5)	**0.003**	0.094	**<0.001**
Time consuming to report^a^	3 (2–4)	3 (2–4)	0.554	3 (2–4)	4 (3–5)	**<0.001**	0.391	**<0.001**
Disrupts the normal workflow^a^	3 (2–5)	3 (2–5)	0.615	3 (2–4)	3 (2–4)	0.178	0.527	0.171
Increases risk of malpractice^a^	3 (2–4)	3 (1–4)	0.153	3 (2–4)	2 (1–3)	**<0.001**	0.652	**<0.001**
Breaks trust with patients^a^	3 (2–3)	3 (2–3)	0.731	3 (2–4)	2 (1–3)	**0.002**	0.305	**<0.001**
Essential knowledge questions								
Are medical doctors obliged to report serious ADRs?	16/32 (50%)	19/36 (53%)	0.819	18/31 (58%)	26/32 (81%)	**0.045**	0.521	**0.013**
Are all serious ADRs known before a drug is marketed?	14/32 (44%)	17/36 (47%)	0.774	18/31 (58%)	29/32 (91%)	**0.003**	0.256	**<0.001**
Where (in The Netherlands) should an ADR report be reported?	22/32 (69%)	26/36 (72%)	0.754	19/31 (61%)	31/32 (97%)	**<0.001**	0.535	**0.006**

Upper part: students’ intentions to report serious, unknown, and all encountered ADRs to the competent authority. Middle part: students’ behavior beliefs toward reporting an ADR. Lower part: students’ knowledge about reporting ADRs (bold: statistical significant difference.

^a^Reported as median and IQR.

#### Student intentions

Before participating in the program, students’ intentions regarding reporting serious ADRs (control median 4, IQR 3–5 vs. intervention median 4, IQR 3–5), previously unrecognized ADRs (control median 4, IQR 3–5 vs. intervention median 4, IQR 3–5), or all ADRs (control median 4, IQR 2–5 vs. intervention median 4, IQR 2–5) were similar. After participating in a J-ADEM team, students were more likely than control students to report serious (pre median 4, IQR 3–5 vs. post median 5, IQR 4–5; *p* = 0.009) and new (pre median 4, IQR 3–5 vs. post median 5, IQR 4–5; *p* = 0.006) ADRs. In contrast, the intentions of students who did not participate in a J-ADEM team did not change significantly. Students who participated in a J-ADEM team were less likely to report all ADRs than were the control group students (control median 4, IQR 3–5 vs. intervention median 3, IQR 2–4; *p* = 0.044) (Table 3).

#### Student attitudes

At the start of the study, all students rated “contributing to medication safety” (control median 4, IQR 3–5 vs. intervention median 4, IQR 3–5) and “improving patient safety” (control median 4, IQR 3–5 vs. intervention median 4, IQR 3–5) as the main reasons to report ADRs. Students were undecided about whether reporting ADRs would “disrupt the normal workflow” (control median 3, IQR 2–5 vs. intervention median 3, IQR 2–5), “break trust with patients” (control median 3, IQR 2–3 vs. intervention median 3, IQR 2–3), or “be time consuming” (control median 3, IQR 2–4 vs. intervention median 3, IQR 2–4). Having participated in the J-ADEM team, students had significantly higher scores in the postintervention questionnaire for attitude regarding ADR reporting and were more aware that ADRs would “contribute to medication safety” (control median 4, IQR 3–5 vs. intervention median 4, IQR 4–5) and “improve patient safety” (control median 4, IQR 3–5 vs. intervention median 5, IQR 4–5) than students who had not participated. J-ADEM team students were also more aware that it is “time consuming to report” (control median 3, IQR 2–4 vs. intervention median 4, IQR 3–5; *p* < 0.001) and that reporting an ADR would not “break trust with patients” (control median 3, IQR 2–4 vs. intervention median 2, IQR 1–3; *p* < 0.001) (Table 3).

#### Student knowledge

More J-ADEM students than control group students were aware that all “serious ADRs should be reported” (control 18/31 vs. intervention 26/32; *p* = 0.045), that not all “serious ADRs are known before a medicine comes on the market” (control 18/31 vs. intervention 29/32; *p* = 0.003), and were “aware where to report an ADR” (control 19/31 vs. intervention 31/32; *p* < 0.001) (Table 3).

#### Student skills in diagnosing ADRs

**Fig. 2:**
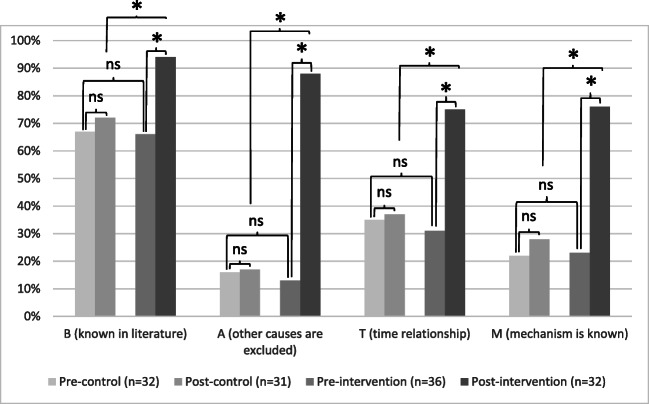
Students’ skills in detecting an adverse drug reaction. Students’ skills in detecting an adverse drug reaction according to the BAT-M method. Percentage of students who would check each of the parameters of the BAT-M when suspecting an ADR. ns = not significant, ^*^p < 0.05

As seen in Fig. 2, most students were unaware of the skills required to diagnose ADRs before participating in a J-ADEM team. While most students would check the current literature (control 67% vs. intervention 67%), only a few students would “exclude other causes” (control 16% vs. intervention 13%), “look for a suitable time relationship” (control 35% vs. intervention 31%), or “check pharmacological mechanisms” (22% vs. intervention 23%). After J-ADEM team participation, students were significantly more skilled at detecting an ADR. More students would consult the current literature (control 72% vs. intervention 94%; *p* = 0.017), “exclude other causes” (control 17% vs. intervention 88%; *p* < 0.001), “look for a suitable time relationship” (control 37% vs. intervention 75%; *p* < 0.001), or “check pharmacological mechanisms” (28% vs. intervention 76%; *p* < 0.001).

## Discussion

This study shows that medical students can play an important role in the detection and management of serious and unknown ADRs in hospitalized patients while at the same time acquiring basic and clinical pharmacovigilance skills and knowledge. The J-ADEM program also provides medical students with the most realistic form of pharmacovigilance training, which is in real clinical practice with maximum perceived responsibility for patient care and the clinical task. Since this training also contributed to clinical practice, we can conclude that J-ADEM team is feasible in a (academic) hospital.

The major advantage of the J-ADEM team approach compared to other physician- and pharmacy-led interventions is that the student team is responsible for the detection, management, and reporting of ADRs. A second advantage is that it saves time and costs. Physicians take on average 30–40 minutes to report an ADR (Sørup et al. 2015), whereas it took physicians less than 5 minutes per patient to supervise the students, which makes the J-ADEM a highly cost-saving initiative. Other clinical educational initiatives involving healthcare students have almost always focused on pharmacy students instead of medical students (Reumerman et al. 2018). Sullivan and Spooner (Sullivan and Spooner 2008) described a comparable and clinically relevant intervention whereby pharmacy students reported already suspected ADRs. While this program led to an increase in ADR reports, it was not organized in a LC-SRC, students did not contribute to the detection of ADRs but only with the documentation, and the study did not evaluate feasibility or describe any learning outcomes. Organizing early clinical involvement at a LC-SRC in the bachelor phase of the curriculum will stimulate students’ intrinsic motivation and self-learning and peer teaching (Schutte et al. 2017c; Schutte et al. 2018b). This will shorten the time to supervise and teach students. We think that this type of learning in a student-run education form should be encouraged.

A previous study from our group looked at medical students who assessed real ADR reports submitted to Lareb (Schutte et al. 2017b). The program improved students’ intentions and attitudes toward ADR reporting and increased their basic pharmacovigilance knowledge and did not cost Lareb staff extra time. While the J-ADEM program yielded similar educational results, it has the advantage that it also significantly increased students’ clinical pharmacovigilance skills in diagnosing an ADR. Additionally, the program was more representative of real-world clinical pharmacovigilance and also increased the number of ADRs reported in hospital.

Our study had some strengths and limitations. The 1-year prospective design allowed us to monitor the opinions of the three stakeholders (students, patients, and physicians) involved with regard to the feasibility of J-ADEM teams. A second strength lies in the case-control design with a preintervention and postintervention questionnaire for evaluating students’ competence in detecting and reporting ADRs. By using a homogeneous control group of active students within the LC-SRC who did not participate in the J-ADEMs team, we tried to limit multiple sources of bias. A third strength lies in the relatively large sample of 159 participants (73 students, 65 patients, and 21 physicians) and high questionnaire response rates (students 76%, patients 86%, and physicians 86%). A final strength lies in the use of previously published questionnaires on ADR reporting, which allowed us to compare the intentions, attitudes, knowledge, and ADR handling capability of healthcare students.

The main limitation of this feasibility study was the relatively short physician survey. Because numerous studies have already extensively evaluated physician competence regarding ADR reporting, we were more interested in establishing whether physicians agreed that the suspected ADR was relevant to report and in learning why they had not reported it themselves. By having a short and quick survey, we hoped we would get more accurate answers and have a high response rate. A second limitation is that we could not determine how often students should participate before they are capable of managing and reporting an ADR themselves. Although the learning benefits in this study were gained by students who participated on average 5.2 times (range 1–9), maximal learning benefits could have been be acquired earlier. A third limitation could be found in the power of some student comparisons. Although we included 73 students in this study, comparisons between the control and intervention group at baseline were underpowered which could mask differences between the groups. A final limitation is the single-center design and the implementation of the J-ADEM teams on only 2 of 23 wards. Because of the aim of the study and the possibility to optimize workflow, we chose to carry out this pilot on only two wards in a single hospital—the internal medicine department with often complex, elderly, polypharmacy patients, high risk for ADRs and the surgical ENT department with often less complex patients using fewer medicines where ARDs are probably less frequent. The clinical outcomes of this study are also monitored and will be reported in a next paper.

Taking these strengths and limitations into account, we conclude that medical students, while learning pharmacovigilance skills and knowledge, can play an important role in the detection and management of ADRs and that a J-ADEM team can be successfully implemented in usual care. The concept of a (student-run) J-ADEM program should be of interest to other universities and indeed in other countries because the detection and reporting of ADRs is a universal problem. As students are keen to participate in our J-ADEM teams and are willing to support physicians in their obligations to report ADRs, the program has the possibility to significantly increase the quantity and quality of ADR reports in a hospital setting at minimal cost. We believe that giving responsibility to students for their learning but also in patient care is the crux of this innovative learning method. However, in this setting of workplace, learning quality control and patient safety needs supervision by a clinical pharmacologist as teacher and as physician. As with many initiatives, this undergraduate training approach will only be successful if postgraduates continue to receive periodic pharmacovigilance training (Herrera 2020; van Eekeren et al. 2018) and are encouraged to detect and report ADRs during ward rounds or during systematic medication reviews. Medication safety will be improved only if undergraduates and postgraduates continue to receive context-based clinical pharmacovigilance education.

## Supplementary Information

ESM 1 Supplementary information: Supplemental original source data (raw data) (XLSX 21 kb)

ESM 2 Supplementary Table 1: Student characteristics (DOCX 12 kb)

## Declarations

### Originality and agreement statement

All of the authors declare that this work has not been, and will not be, published in whole or part in any other journal and agree to the contents of the manuscript in its submitted form.
