# Supplementary material for: Medical students as adverse drug event managers, learning about side effects while improving their reporting in clinical practice
Source: Naunyn Schmiedebergs Arch Pharmacol. 2021 Mar 5;394(7):1467–76. doi: 10.1007/s00210-021-02060-y (PMC8233281; doi:10.1007/s00210-021-02060-y)
Supplement: Supplementary file 2 — Supplementary Table 1: Student characteristics (DOCX 12 kb) [file 210_2021_2060_MOESM2_ESM.docx]

| Student characteristics | | Control group (n=32) | Intervention group (n=36) |
| --- | --- | --- | --- |
| Gender | | | |
|  | Female (%) | 20 (63) | 24 (67) |
| Years of medical school | | | |
|  | First year medical student (%) | 3 (9) | 2 (6) |
|  | Second year medical student (%) | 8 (25) | 11 (31) |
|  | Third year medical student (%) | 8 (25) | 14 (39) |
|  | Fourth year medical student (%) | 9 (28) | 7 (19) |
|  | Fifth year medical student (%) | 3 (9) | 2 (6) |
|  | Sixth year medical student (%) | 1 (3) | - (-) |

Supplemental table 1, Student characteristics of gender and year of medical school.
